# *Salpianthus macrodontus* Extracts, a Novel Source of Phenolic Compounds with Antibacterial Activity against Potentially Pathogenic Bacteria Isolated from White Shrimp

**DOI:** 10.3390/molecules27144397

**Published:** 2022-07-08

**Authors:** Pedro Ulises Bautista-Rosales, Alexeyevich Jassiel Prado-Murguía, Iza Fernanda Pérez-Ramírez, Rosalía Servín-Villegas, Francisco Javier Magallón-Barajas, Rosendo Balois-Morales, Verónica Alhelí Ochoa-Jiménez, Paola Magallón-Servín

**Affiliations:** 1 Programa de Maestría en Ciencias Biológico Agropecuarias, Universidad Autónoma de Nayarit, Km. 9 Carretera Tepic-Compostela, Xalisco 63780, Nayarit, Mexico; ubautista@uan.edu.mx (P.U.B.-R.); alex_ajpm@hotmail.com (A.J.P.-M.); rmbalois@uan.edu.mx (R.B.-M.); 2 Unidad de Tecnología de Alimentos, Secretaría de Investigación y Posgrado, Universidad Autónoma de Nayarit, Ciudad de la Cultura S/N, Colonia Centro, Tepic 63000, Nayarit, Mexico; veronica.ochoa@uan.edu.mx; 3 Facultad de Química, Universidad Autónoma de Querétaro, C.U., Cerro de las Campanas S/N, Santiago de Queretaro 76010, Queretaro, Mexico; iza.perez@hotmail.com; 4 Centro de Investigaciones Biológicas del Noroeste, Km. 1 Carretera a San Juan de La Costa “El Comitan”, La Paz 23205, Baja California Sur, Mexico; rservin04@cibnor.mx (R.S.-V.); fmagallon04@gmail.com (F.J.M.-B.); 5 Centro de Investigaciones Biológicas del Noroeste as Part of the Program of Catedras CONACyT, Km. 1 Carretera a San Juan de La Costa “El Comitan”, La Paz 23205, Baja California Sur, Mexico; 6 Bashan Institute of Sciences, 1730 Post Oak Ct., Auburn, AL 36830, USA

**Keywords:** minimum inhibition concentration, bacterial motility, biofilm formation, polyphenolic profile, aquaculture

## Abstract

This study aimed to evaluate the antibacterial activity in vitro of *Salpianthus macrodontus* and *Azadirachta indica* extracts against potentially pathogenic bacteria for Pacific white shrimp. Furthermore, the extracts with higher inhibitory activity were analyzed to identify compounds responsible for bacterial inhibition and evaluate their effect on motility and biofilm formation. *S. macrodontus* and *A. indica* extracts were prepared using methanol, acetone, and hexane by ultrasound. The minimum inhibitory concentration (MIC) of the extracts was determined against *Vibrio parahaemolyticus, V. harveyi, Photobacterium damselae* and *P. leiognathi*. The polyphenol profile of those extracts showing the highest bacterial inhibition were determined. Besides, the bacterial swimming and swarming motility and biofilm formation were determined. The highest inhibitory activity against the four pathogens was found with the acetonic extract of *S. macrodontus* leaf (MIC of 50 mg/mL for *Vibrio* spp. and 25 mg/mL for *Photobacterium* spp.) and the methanol extract of *S. macrodontus* flower (MIC of 50 mg/mL for all pathogens tested). Both extracts affected the swarming and swimming motility and the biofilm formation of the tested bacteria. The main phenolic compounds related to *Vibrio* bacteria inhibition were naringin, vanillic acid, and rosmarinic acid, whilst hesperidin, kaempferol pentosyl-rutinoside, and rhamnetin were related to *Photobacterium* bacteria inhibition.

## 1. Introduction

The Pacific white shrimp (*Litopenaeus vannamei*) is globally one of the most important aquaculture species [1]. The shrimp aquaculture industry was intensified between 2002 and 2012; however, the intensification caused different sanitary problems related to mortality caused by bacteria, such as *Vibrio* spp., challenging productivity, and survival intensive farms [2,3,4]. Among the main causative agents of bacterial diseases in shrimp are *Vibrio angullarum*, *V. ordalii*, *V. salmonicida*, *V. vulnificus*, *V. alginolyticus*, *V. harveyi*, *V. ponticus*, *V. parahaemolyticus*, *V. mimicus*, *Photobacterium damselae*, and *P. leiognathi* [5,6,7,8].

Shrimp farmers use, prophylactically or therapeutically, a wide variety of antibiotics to control diseases. The application of commercial antibiotics is permitted to control bacterial infectious disease in aquaculture organisms, primarily using enrofloxacin, florfenicol, oxytetracycline, sarafloxacin, fosfomycin, monensin, salinomycin, and semduramicin [9,10]. However, their inefficient and unsafe use has contributed to the appearance of resistant bacteria in aquaculture systems and natural coastal marine systems [9,11]. Some reports show the resistance of *Vibrio* sp. (isolated from shrimp farms) towards different antibiotics, such as *V. coralliiyticus* [12], *V. parahaemolyticus* associated with acute hepatopancreatic disease (AHPND) [13], as well as *V. navarrensis*, *V. brasilensis*, *V. xuii*, *V. alginolyticus*, *V. cholera*, *V. vulnificus* [9,14]. Due to the above, antibiotic resistance of *Vibrio* spp. is a major health problem, and it is necessary to use non-antibiotic strategy as the control method of these bacteria [14]. As a result, international sanitary agencies, including FAO, recommend controlling antibiotics and using non-antibiotic therapy [15].

When potential pathogenic bacteria present antibiotic resistance, one approach could be exploited: the search for new antimicrobials to be safely used in aquaculture. In the search of these antimicrobials, three aspects are necessary to address: (1) those that are non-specific (can affect different types of potentially pathogenic bacteria), (2) those that are produced by low-impact industry (no industrialization necessary) for environmental reasons, and (3) those that can have an effect on biofilm formation (associated with high antibiotic resistance). Among these antimicrobials are the plant extracts, which contain natural compounds, such as phenolic compounds, polysaccharides, and proteoglycans, which could stimulate the immune system and prevent or control infectious microorganisms [16]. Although the antimicrobials cannot be used in the farm ponds water, there is a huge potential to be used as additives in feeds, supplements, and cleaning agents in nurseries (where chemicals are not recommended).

In that regard, extracts of *Azadirachta indica* A. Juss (Meliaceae) and *Salpianthus macrodontus* (Nyctaginaceae) extracts are used in some regions of Nayarit, Mexico, as products with ethnomedicinal compounds with relevant antimicrobial activity, which can be employed in aquaculture as antibacterial agents in novel application methods. *A. indica* extracts have been reported as *Vibro* spp. inhibitors. Their minimum concentration inhibitory may vary in a range of 0.075 mg/mL to 250 mg/mL depending on the nature of the extract and the strain studied [17,18,19,20,21,22]. On the other hand, *S. macrodontus* extracts have been reported as an inhibitor of fungi, such as *Penicillium chrysogenum* and *P. expansum* [23], however, there is no report so far about their use to control bacteria or to be used as antibacterials.

Therefore, the aim of this study was to evaluate the antibacterial activity in vitro of extracts from *S. macrodontus* and *Azadirachta indica* against potentially pathogenic bacteria for Pacific white shrimp, determining their minimum inhibitory concentration (MIC) and their impact on swimming, swarming motility, and biofilm formation. Furthermore, for this study we also aimed to determine the phenolic profile of the plant extracts and what were the active compounds that presented the higher antibacterial activity against the pathogenic bacteria tested in this work.

## 2. Results

### 2.1. Antibacterial Susceptibility Assay and Minimum Inhibitory Concentration (MIC)

**Figure 1:**
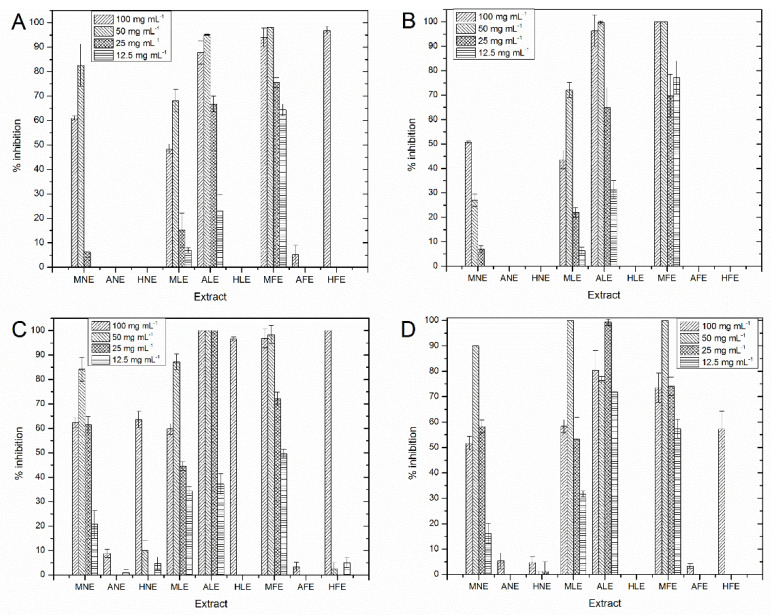
Inhibition percentage of the extracts at different concentrations against (**A**) *V. parahaemolyticus*; (**B**) *V. harveyi*; (**C**) *P. damselae*; (**D**) *P. leiognathi*.

Table 1 shows the maximum inhibition percentage and the MIC at the evaluated conditions. The extracts obtained from the leaves and flowers of *S. macrodontus* showed higher antibacterial activity against the bacteria tested (*p* < 0.05) than the *A. indica* extracts. Figure 1 shows that at higher extract concentrations, the inhibition rate is greater in most cases. However, the highest concentration was not always the most effective to inhibit bacteria.

The MICs of ALE (acetone leaves extract) were 50 mg/mL against *V. parahaemolyticus* and *V. harveyi*, 25 mg/mL against *P. damselae*, and *P. leiognathi*. The MIC to MFE (methanol flower extract) was 50 mg/mL for all bacteria evaluated (Table 1). From the *A. indica* extracts, only the MNE (methanol leaves of *A. indica* extract) was effective against the four bacteria evaluated in 50 and 100 mg/mL concentrations. However, several extracts obtained from the leaves and flowers of *S. macrodontus* were better (*p* < 0.05) than the MNE (Figure 1). In this regard, at a 100 mg/mL concentration, HLE (hexane leaves extract) was better against *P. damselae*, and HFE (hexane flowers extract) was better against *V. parahaemolyticus* and *P. damselae*. Due to the above, ALE and MFE were chosen for the following tests. Therefore, ALE and MFE extracts can be used to treat infectious diseases caused by resistant pathogens. This is the first report of the antibacterial activity of *S. macrodontus*.

### 2.2. Analysis of Polyphenolic Compounds of Extracts

The polyphenolic profiles of the *S. macrodontus* flowers and leaves extracts are shown in Table 2. A total of 45 compounds were identified, of which 27 were flavonoids, and 18 were phenolic acids.

Flavonoids were classified in flavanols (3 compounds), flavanones (6 compounds), and flavonols (18 compounds). Simultaneously, the phenolic acids were identified as hydroxybenzoic acids (6 compounds) and hydroxycinnamic acids (12 compounds).

The MLE (methanol leaves extract) showed the highest concentration of the phenolic compounds (1017.34 mg/mL), followed by MFE (253.72 mg/mL), and then ALE (215.35 mg/mL). Quercetin hexoside stands out as the phenolic compound with the highest concentration in the MLE, followed by quercetin hexoside-rhamnoside, kaempferol dihexoside, kaempferol rutinoside, and kaempferol hexosyl-rhamnosyl-hexoside. The main compounds found in ALE were kaempferol dihexoside, quercetin hexoside, eriocitrin, and kaempferol rutinoside. On the other hand, the main compounds in MFE were kaempferol hexosyl-rhamnosyl-hexoside, followed by quercetin hexoside-rhamnoside, quercetin dihexoside, kaempferol trihexoside, and kaempferol dihexoside.

### 2.3. Effect of Polyphenols on the Bacterial Inhibition

**Figure 2:**
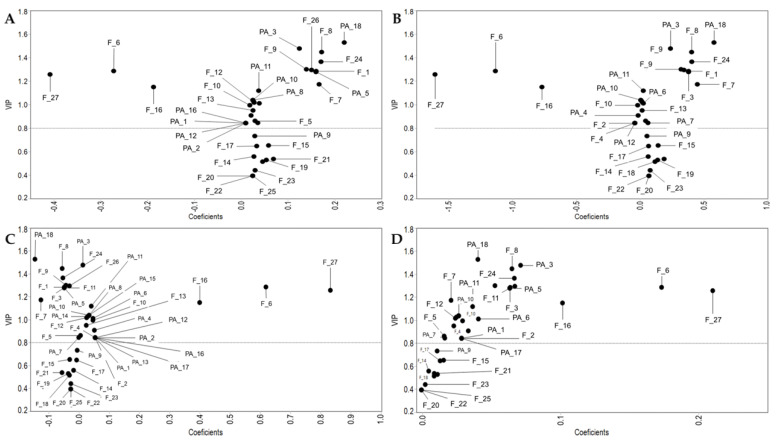
Association of polyphenols of S. macrodontus extracts and microbial growth. (**A**) *V. parahaemolyticus*, (**B**) *V. harveyi*, (**C**) *P. damselae*, and (**D**) *P. leiognanathi*.

Figure 2 shows PLS-DA plots constructed with polyphenol profile of extracts and inhibition percentage. The preceding is in order to identify the bioactive compounds associated with the inhibition of the different bacteria evaluated. Those compounds with VIP > 0.8 and coefficient values > 0 can be considered responsible for the extracts’ inhibitory activity. Likewise, compounds with VIP > 0.8 and coefficient values < 0 can be regarded as growth stimulators.

The effects of the compounds on the growth of *V. parahaemolyticus* and *V. harveyi* are shown in Figure 2A,B, respectively. Both bacteria had a similar response to phenolic compounds. Naringin (F_8), vanillic acid (PA_3), and rosmarinic acid (PA_18) were the compounds mainly related to the inhibition of *Vibrio* bacteria since they had higher VIP and coefficient values. Naringin was found in the methanolic and acetone extracts of *S. macrodontus* flowers and leaves. Vanillic acid was found in MFE, AFE (acetone flower extract), and ALE. Rosmarinic acid was found in all the evaluated extracts, except HLE.

Moreover, Figure 2C,D show the compounds related to inhibition of *P. damselae* and *P. leiognathi*, respectively. Hesperidin (F_6), kaempferol pentosyl-rutinoside (F_16), and rhamnetin (F_27) are mainly related to inhibition of *Photobacterium* bacteria since they had higher VIP and coefficient values. Hesperidin was found in all evaluated extracts. kaempferol pentosyl-rutinoside was found in all evaluated extracts except HFE. Rhamnetin was presented in the acetone and hexene extracts of flower and leaf.

It is important to note that the three main compounds that inhibit the growth of *Photobacterium* bacteria (hesperidin (F_6), kaempferol pentosyl-rutinoside (F_16), and rhamnetin (F_27)), enhanced the growth of *Vibrio* bacteria (Figure 2).

### 2.4. Motility Assays

**Figure 3:**
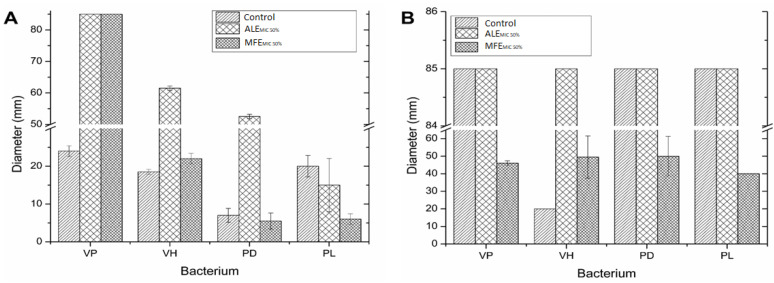
Effect of the extracts on *V. parahaemolyticus* (VP), *V. harveyi* (VH), *P. damselae* (PD), and *P. leiognathid* (PL) motility. (**A**) Swarming and (**B**) swimming motility.

The result obtained from the motility assays of ALE and MFE extracts (Figure 3) showed significant differences among four bacteria and control (without extract) (*p* < 0.05). The MFE extract had a higher effect against the four bacterial strains tested for both types of motilities.

The ALE increased the swarming motility of *V. parahaemolyticus*, *V. harveyi*, and *P. damselae* on 254.17, 232.43, and 650%, respectively. In contrast, *P. leiognathi* had a reduction of 25%. On the other hand, MFE increased the swarming motility of *V. parahaemolyticus*, *V. harveyi*, and *P. damselae* by 87.5, 18.92, and 21.42%, respectively. However, *P. leiognathi* decreased its swarming motility by 70% in the presence of MFE (Figure 3A).

Furthermore, ALE did not change the swimming motility for *V. parahaemolyticus*, *P. damselae*, and *P. leiognath*, while *V. harveyi* increased 325% regarding control. On the other hand, MFE decreased by 45.88, 41.18, and 52.94% in *V. parahaemolyticus*, *P. damselae*, and *P. leiognathi*, respectively, while *V. harveyi* increased the swimming motility 147.5% concerning control (Figure 3B).

### 2.5. Microplate Assay for Biofilm Quantification

In this study, statistical analysis indicated a significant difference in ALE and MFE on the biofilm formation of these bacteria (*p* < 0.05). ALE reduced biofilm formation on 69.25, 100, and 61.13% in *V. parahaemolyticus*, *V. harveyi*, and *P. leiognathi*. However, *P. damselae* did not form biofilm. Furthermore, the MFE did not affect significantly the biofilm formation in *V. parahaemolyticus* and *P. leiognathi* (*p* > 0.05); however, *V. harveyi* increased the biofilm formation 372.4% (*p* < 0.05).

## 3. Discussion

Several plant extracts and essential oils have been used to control pathogenic bacteria in aquaculture (*Vibrio* and *Photobacterium* bacteria), such as boiled water extract of *Psidium guava* leaf, green tea leaf, and water and oil extracts of *Calendula officinalis* [19,20], *Piper betle* ethyl acetate [24], and *Scutellaria baicalensis* water extract [24]. This tendency has responded for the need of new antimicrobials that can be obtained from a low-impact technology (reduction in solvents, industrial process, residuals) and that contain different active ingredients to reduce the development of resistance.

In the case of *A. indica*, there are several studies where extracts of this plant have been effective in the control of genus *Vibrio*, including *V. parahaemolyticus* at concentrations of 0.1 to 100 mg/mL [17,18,19,20,21,22], *V. alginolyticus* at concentrations of 0.075 mg/mL to 250 mg/mL [17,18], and *V. cholerae* at concentrations of 0.1 mg/mL to 15 mg/mL [22]. Banerjee [17] found an MIC of 3.13% (equivalent to 31.3 mg/mL) of *A. indica* juice, which is lower than that shown in the present study. Moreover, the aqueous extract of leaves from *A. indica* (MIC of 10 mg/mL) reported by Sharma and Patel [22], besides the ethanol, methanol, chloroform, and acetone extracts from the leaves of *A. indica* (MIC of 0.1, 0.25, 0.075, and 0.25 mg/mL, respectively) reported by Dhayanithi et al. [18]. However, in the present study, *A. indica* extracts did not completely inhibit the four bacteria tested. The bacteria probably developed a differential resistance against this plant extract in a similar manner as some *Vibrio* strains have shown resistance to antibiotics [9,12,13,14]. Pathogenic bacteria can become resistant to antibacterial agents through mutation and selection or by acquiring genetic information that encodes other bacteria [25].

In this study, we established the novel use of *S. macrodontus* extracts’ efficacy against Vibrionaceae family bacteria tested, indicating that the plant produces compounds that affect the bacterial defense mechanisms. The antimicrobial activity of both ALE and MFE showed inhibition of *Vibrio* species was similar (MIC 50 mg/mL). However, ALE demonstrated a higher capacity to inhibit *Photobacterium* species (MIC of 25 mg/mL) than MFE (MIC of 50 mg/mL). In both extracts, MIC does not suggest a dose–response relationship, however, a high dose of the extract can stimulate the growth of bacteria causing a hormesis effect. Therefore, in both extracts, phytochemical compounds probably exert a differential effect based on their active compounds as we can observe in Table 2.

We found flavonoids, such as flavanols, flavanones and flavonols, as well as phenolic acids, such as hydroxybenzoic acids and hydroxycinnamic acids, as the main components of *S. macrodontus* extracts. Daglia [26] mentions that flavanols and flavonols have a wide spectrum and higher antimicrobial activity than other polyphenols since they can suppress virulence factors, such as biofilm formation inhibition and the reduction in host ligands adhesion, and the neutralization of bacterial toxins.

In this work, the main compounds related to *Vibrio* bacteria inhibition were Naringin (F_8), vanillic acid (PA_3), and rosmarinic acid (PA_18). Furthermore, the compounds hesperidin (F_6), kaempferol pentosyl-rutinoside (F_16), and rhamnetin (F_27) were related to the inhibition of *Photobacterium* bacteria. These six compounds have been reported as antibacterial compounds. Naringin has shown inhibitory action against a wide-spectrum of Gram-positive and Gram-negative bacteria [26]. Vikram et al. [27] proved that different flavonoids, such as naringin, kaempferol, quercetin and epigenin, affected *V. harveyi* biofilm formation and virulence (genes encoding TTSS).

Vanillic acid has been proven to have antibacterial effects against *E. coli*, *Pasteurella multocida*, *Neisseria gonorrhoeae*, *Klebsiella pneumoniae*, and *Staphylococcus aureus*, possibly to increased membrane permeability and antibiotic accumulation in pathogens [28,29,30]. Liu et al. [31] proved that vanillic acid presents an antibacterial and an antivirulence effect on *Vibrio alginolyticus*, with a MIC of 1 mg mL^−1^. The vanillic acid effect on *V. alginolyticus* causes cell membrane damage and increasing membrane permeability and affects biofilm-forming capability, mobility and exotoxin production.

Rosmarinic acid has shown antibacterial activity against *Pseudomona aureaginosa* and *E. coli* due to their strong cytotoxic potency and genotoxic effects [32,33,34,35]. This activity is related to enzyme inhibition by oxidized compounds due to reactions with sulfhydryl groups of non-specific interactions with proteins [36]. Corrales et al. [37] and Khalid et al. [27] reported that hesperidin shows inhibitory action against a wide-spectrum of Gram-positive and Gram-negative bacteria, which is related to bacterial membrane disruption and interference with microbial enzymes [36]. Biharee et al. [38] and Daglia [26] reported that rhamnetin has antimicrobial activity against Gram-positive, *Candida albicans*, and *Chlamidia pneumoniae*. Rhamnetin can cause membrane disruption [38] and decrease the infective yields and the compounds related to pathogenesis [26]. Furthermore, Cid-Ortega and Monroy-Rivera [39] and Sati et al. [40] mention that kaempferol glycosides, such as kaempferol pentosyl-rutinoside, have antibacterial activities against Gram-positive and Gram-negative bacteria.

Also, we observed that hesperidin (F_6), kaempferol pentosyl-rutinoside (F_16), and rhamnetin (F_27)) inhibited *Photobacterium bacteria* but enhanced the growth of *Vibrio bacteria*. This can be related to the hormesis phenomenon, where stimulatory responses (bacterial growth) occur at low doses of antibacterial compounds. In contrast, inhibitory responses (antibacterial activity) appear at higher doses, which form a dose–response relationship [41]. These three compounds could be in enough doses to inhibit Photobacterium bacteria but not enough to inhibit Vibrio bacteria.

On the other hand, ALE and MFE showed antipathogenic activities since they affected the virulence factors, such as motility (swarming and swimming) and the biofilm formation capacity of bacteria.

Both extracts (ALE and MFE) increased the swarming motility of *V. parahaemolyticus*, *V. harveyi*, and *P. damselae*, and swimming motility to *V. harveyi*. Increased motility in bacteria (chemotaxis) could respond to avoiding contact with the antimicrobial compounds present in the extracts [42]. On the other hand, *P. leignathi* decreased its swarming motility in the presence of ALE and MFE, as well as *V. parahaemolyticus*, *P. damselae*, and *P. laiognathi* decreasing their swimming motility in the presence of MFE at sublethal doses. One of the responsible compounds for this phenomenon could be naringin, which was reported as an inhibitor of swimming and swarming motility in *Chromobacterium violaceum* and *Yersinia enterocolitica* [32,34]. This could be due to the inhibition of the microorganisms in question or directly affected by the bacteria flagella [42].

Regarding the virulence factor biofilm production, ALE reduced biofilm production, even using 50% of the minimum inhibitory concentration. This reduction can be because of the naringin, rosmarinic acid, and hesperidin, which have been reported as inhibitors in biofilm production [32,34,35,43,44,45]. Santhakumari and Ravi [44] mention that naringin interferes with the acyl homoserine lactone-based QS of a wide range of Gram-negative bacteria, which is related to biofilm production.

However, MFE had low control of biofilm formation capacity or even increased its production. This may be because the dose was inadequate for the bacteria tested since MFE showed a lower concentration of the main antibacterial compounds than ALE. The low concentration of antibacterial compounds with reports of antibiofilm activity, such as naringin, rosmarinic acid, and hesperidin, can cause stress and induce biofilm production to protect themselves from toxic substances [26].

## 4. Materials and Methods

### 4.1. Plant Material

Leaves and flowers of *S. macrodontus* were collected near Tuxpan, Nayarit, Mexico (21°56′7.1808” N 105°15′28.584” W). In contrast, *Azadirachta indica* leaves were collected from La Paz, Baja California Sur, Mexico (24°8′10.346” N 110°25′36.431” W). Both plants were identified by José Juan Perez Navarro, a researcher from the Centro de Investigaciones Biológicas del Noroeste, S. C. (CIBNOR) in Baja California Sur, Mexico, and corroborated by Ana Maria Hanan Alipi, a researcher from the Universidad Autónoma de Nayarit (UAN) in Xalisco, Nayarit, México. *S. macrodontus* was kept in the Herbarium of Investigación y Posgrado of Universidad Autonóma de Nayarit under the A. Hanan 3765 id. The vegetal material was dried in a 12 L lyophilization system with stoppering tray dryers (LABCONCO Freeze Dry System Freezone) at −40 °C in vacuum conditions. Finally, the dried plants were ground with a coffee grinder.

### 4.2. Preparation of the Vegetable Extracts

For the preparation of the extracts, a solvent was added in a 1:10 ratio (dry sample: solvent) and sonicated in an ultrasonic bath Branson^®^ 5510 (47 kHz at 130 W) for 30 min at ≤40 °C [46]. In this way, the methanol, acetone, and hexane extracts were obtained from *A. indica* leaves and *S. macrodontus* leaves and flowers. Then, the supernatant was recovered by vacuum filtration (40 Torr) through Whatman paper No. 1, and the solvent was eliminated in a rotary evaporator (Büchi R-3) at no more than 40 °C under vacuum conditions (40 Torr). Afterward, the extracts were resuspended in glycerol at 20% until reaching a concentration of 1 g/mL. Then, the extracts were dissolved in the same manner in tryptic soy broth at 50% (TSB 50%) supplemented with 20 g/L of sodium chloride (TSB20 50%) in order to get a final concentration of 100 mg/mL (stock solution). Finally, all extracts were sterilized using filters with a pore size of 0.22 μm and stored at −20 °C until later analysis to avoid denaturation.

The extracts were named by three characters. The first one means the solvent used (M for methanol, A for acetone, and H for Hexane). The second character is according to the vegetable source from which it was obtained (L for *S. macrodontus* leaves, F for *S. macrodontus* flowers, and N for *A. indica* leaves). The last character means extract.

### 4.3. Bacterial Strains

Four pathogenic strains, previously isolated from white shrimp showing signs of AHPND in Mexico shrimp farms (2013), were used for the susceptibility analysis. These bacteria were kindly provided from the CIBNOR collection (Environmental Microbiology Group, CIBNOR, La Paz, México). The strains used for this study were: *Vibrio parahaemolyticus* 2, *Vibrio harveyi* 6F, *Photobacterium damselae* 7F, and *Photobacterium leiognathi* 8F.

### 4.4. Antibacterial Assay and Minimum Inhibitory Concentration (MIC)

Antibacterial activity and MIC were determined based on the broth microdilution technique described by the Clinical and Laboral Standards Institute (CLSI) [47], with minor modifications. In order to do this, serial dilutions of the extracts were made with TSB20 50% to obtain a concentration range of 12.5–100 mg/mL from the stock solutions. Later, 150 µL of each culture medium was added to the microplate wells, followed by 10 µL of a bacterial suspension (0.4 optical density at 620 nm, which corresponds to 1 × 10^8^ cells/mL) of the strain to be evaluated. Afterward, the plates were incubated for 20 h at 35 °C, and the optical density at 620 nm was recorded with a microplate reader (Thermo Scientific). Finally, the inhibition percentage was calculated of the extracts tested concerning the control (no extract). In the present study, the MIC was taken as the extract concentration that reduced the bacteria growth between 95 and 100%, according to CLSI (Clinical and Laboratory Standards Institute) [47].

### 4.5. Polyphenol Profile by UPLC-ESI-Q-ToF MS

Samples of the concentrated plant extract (200 mg) were dissolved in 8 mL of methanol: water (50:50 *v*/*v*) acidity with HCl (pH 2); next, it was thoroughly shaken at room temperature for 1 h, it was centrifugated at 16,000× *g* for 10 min at 4 °C. The supernatant was recovered. A total of 20 mL of acetone/water (70:30 *v*/*v*) was added to the residue. The shaking and centrifugation were repeated. The methanol and acetone extracts were mixed and filtered through PVDF syringe filters (13 mm, 0.45 µm).

An aliquot (1 mL) of the phenolic extract was evaporated to dryness (Speedvac, Savant, Thermo Fisher Scientific, MA, USA) and was resuspended in 200 µL of methanol. Then, it was filtered (0.45 µm). The polyphenol profile was analyzed using an ultra-performance liquid chromatography system (UPLC) Acquity UPLC ™H-Class (Waters, Manchester, UK) coupled to a mass spectrometer quadrupole-time of flight (MS QTof) with an atmospheric pressure electrospray ionization (ESI) interface (Vion, Waters Co, MA, USA). The column used was an Acquity BEH C18 (100 × 2.1 mm, 1.7 um) at 35 °C.

The elution gradient was performed with a binary system consisting of (A) 0.1% formic acid in water and (B) 0.1% formic acid in acetonitrile. The following gradient was applied at a flow rate of 0.4 mL/min: 0 min at 0% B, 2.5 min at 15% B, 10 min at 21% B, 12 min at 90% B, 13 min at 95% B; 15 min at 0% B, and 17 min 0% B. The injection volume was 2 μL, and the sample temperature was set at 10 °C.

The Q-ToF MSE conditions were as follows: data were acquired at negative ionization (ESI-) within a mass range of 100 to 1200 Da; capillary voltage, 2.5 kV (ESI-) and 3.5 kV (ESI+); cone voltage, 40 eV; low collision energy, 6V. The conditions of the mass spectrometer were as follows: the temperature of the source was adjusted to 120 °C and nitrogen was used as the desolvation gas (800 L/h) at a temperature of 450 °C. The sampling cone was 40 eV, and capillary voltages were 2.0 kV (ESI-) and 3.5 kV (ESI+). Data acquisition was performed using the high definition MSE negative ionization mode with a 50–2000 Da mass range. Leucine-enkephalin (50 pg/mL) at 10 mL/min was used for mass correction. Peak identification was carried out by identifying the exact mass of the pseudo-molecular ion (mass error < 5 ppm), isotope distribution, and fragmentation pattern. Calibration curves were constructed with ellagic acid (hydroxycinnamic acids), gallic acid (hydroxybenzoic acids), (-)-epicatechin (flavanols), naringenin (flavanones), and quercetin (flavonols). Data acquisition was performed with the UNIFI Scientific Information System (Waters Co., MA, USA). The extracts were analyzed in triplicate.

### 4.6. Motility Assays

Swimming (flagella-directed movement in aqueous environments) and swarming (flagella-directed rapid movement onto solid surfaces) assays were performed as described by de la Fuente-Núñez et al. [48] with some modifications. Briefly, individual colonies were transferred from TSB20 agar to the surface of swimming agar (0.3% Difco Bacto Agar) and swarming agar (0.5% Difco Bacto Agar) using a sterile sharp toothpick. After incubation at 35 °C for 20 h, the motility was assessed by measuring the distance the bacteria had moved off the inoculation point, expressed as diameter (mm).

### 4.7. Microtiter Plate Assay for Biofilm Quantification

Biofilm formation assays were performed according to Naves et al. [49] with some modifications. A volume of 10 µL of inoculum with 0.4 OD620 was inoculated in 200 µL of tryptic soy broth (TSB 20%) containing 20 g/L of sodium chloride was added in peripheral wells. Then, the microplate was incubated for 20 h at 35 °C (without agitation). After, the biofilms were fixed with a crystal violet solution (1%) for 15 min. Then, the excess crystal violet dye was removed with water, plates were washed twice, and air-dried. At that point, 200 μL of 95% ethanol was added to all well and kept in orbital shaking (130 rpm) for 18 h. Finally, biofilm measurements were determined using Equation (1).

(1) $$\mathrm{SBF}=\frac{\left(\mathrm{AB}-\mathrm{CW}\right)}{G}$$

where SBF is the specific biofilm formation, AB is the OD540 of the attached and stained bacteria, CW is the OD540 of the control medium (no bacteria), and G is the microbial growth before crystal violet staining (OD620). The spectrophotometric measures were obtained using a microplate reader (Thermo Fisher Scientific Mutiskan Go, Vantaa, Finland). The SBF values were classified into two categories: strong biofilm producers (SBF index 1.00) and weak biofilm producers (SBF index 1.00).

### 4.8. Statistical Analysis

The results were expressed as means ± SD. Each extract was tested in triplicate in three independent experiments. Statistical significance of the differences between means was established by testing homogeneity of variance and normality of distribution followed by ANOVA with Tukey test (analysis of classes of phytochemical compounds). The non-parametric methods (Kruskal–Wallis test) were used for the antibacterial activity of extracts. The *p* values below 0.05 were considered statistically significant. All analyses were performed using SAS software version 9.4 for Windows.

Associations between the polyphenolic compounds and inhibition (%) were assessed with the Variable Importance in the Projection (VIP) vs. coefficient score plots constructed from the supervised Partial Least Squares-Discriminant Analysis (PLS-DA) with centered and scale data. A non-linear iterative partial least squares (NIPALS) was used. This analysis was carried out with JMP software (v10) (Sytat Software, Inc., San José, CA, USA).

## 5. Conclusions

The present study shows the potential antibacterial activity of *S. macrodontus* ALE and MFE against shrimp pathogens. The ALE and MFE antimicrobial potential against evaluated Vibrio bacteria is mainly due to naringin, vanillic acid, and rosmarinic acid, while against photobacterium bacteria is mainly due to hesperidin, kaempferol pentosyl-rutinoside, and rhamnetin. The ALE and MFE showed antipathogenic activity modifying the speed of motility (swarming and swimming) and biofilm formation, which could be related to compounds present in extracts, mainly naringin, rosmarinic acid, and hesperidin.

## 6. Patents

The patent MX 391053 B resulted from the work reported in this manuscript.

## Figures and Tables

**Table 1:** Maximum antibacterial activity of the obtained extracts and their minimum inhibitory concentration.

Extract Key	Maximum Inhibition %				MIC (mg/mL)			
	VP	VH	PD	PL	VP	VH	PD	PL
MNE	82.63	50.70	84.19	89.96	-	-	-	-
ANE	NP	NP	8.81	5.36	-	-	-	-
HNE	NP	NP	63.57	4.76	-	-	-	-
MLE	68.12	72.10	87.26	100 *	-	-	-	50 *
ALE	95.08 *	99.66 *	100 *	99.27 *	50 *	50 *	25	25
HLE	NP	NP	96.51 *	NP	-	-	100 *	-
MFE	98.21 *	100 *	98.28 *	100 *	50 *	50 *	50	50 *
AFE	5.16	NP	3.32	3.30	-	-	-	-
HFE	96.88 *	NP	100 *	57.23	-	-	100 *	-

The averages marked by an asterisk (for each bacteria) are statistically equal according to the Kruskall–Wallis (*p* > 0.05) test. *V. parahaemolyticus* (VP), *V. harveyi* (VH), *P. damselae* (PD), and *P. leiognathi* (PL). Methanol (M), Acetone (A), Hexane (H), flower of *S. macrodonus* (F), leaves of *S. macrodonus* (L), leaves of *A. indica* (N).

**Table 2:** Polyphenolic profile of *S. macrodontus* extracts.

Family	Code	Component Name	Retention Time (min)	Molecular Formula	Expected Mass (Da)	Observed *m*/*z*	Mass Error (ppm)	Adducts	Concentration (µg/mL)					
									MFE	AFE	HFE	MLE	ALE	HLE
Flavanols	F_1	(Epi)-catechin hexoside	1.54	C_21_H_24_O_11_	452.1319	451.1229	−3.6505	[M-H]^−^	0.08 ± 0.00	2.76 ± 0.03	ND	ND	9.97 ± 0.07	ND
	F_2	(Epi)-catechin^+^	2.59	C_15_H_14_O_6_	290.0790	289.0720	0.8901	[M-H]^−^	0.21 ± 0.00	ND	ND	ND	ND	ND
	F_3	(Epi)-catechin gallate	3.05	C_22_H_18_O_10_	442.0900	441.0829	0.4607	[M-H]^−^	ND	ND	ND	ND	0.21 ± 0.00	ND
Flavanones	F_4	Eriodictyol	5.56	C_15_H_12_O_6_	288.0634	287.0564	0.9815	[M-H]^−^	0.06 ± 0.00	0.02 ± 0.00	ND	ND	ND	ND
	F_5	Naringenin hexoside	5.80	C_21_H_22_O_10_	434.1213	433.1148	1.9102	[M-H]^−^	0.25 ± 0.00	ND	ND	0.41 ± 0.00	ND	ND
	F_6	Hesperidin	6.34	C_28_H_34_O_15_	610.1898	609.1830	0.9104	[M-H]^−^	0.58 ± 0.03	0.26 ± 0.01	0.31 ± 0.00	10.19 ± 0.30	13.61 ± 0.13	0.63 ± 0.01
	F_7	Naringenin^+^	10.97	C_15_H_12_O_5_	272.0685	271.0604	−2.8797	[M-H]^−^	4.16 ± 0.09	7.77 ± 0.16	0.02 ± 0.00	0.34 ± 0.00	1.18 ± 0.00	ND
	F_8	Naringin	12.85	C_27_H_32_O_14_	580.1792	579.1710	−1.5524	[M-H]^−^	0.67 ± 0.00	5.05 ± 0.07	ND	2.55 ± 0.20	3.05 ± 0.00	ND
	F_9	Eriocitrin	12.98	C_27_H_32_O_15_	596.1741	595.1670	0.3245	[M-H]^−^	10.04 ± 0.12	1.34 ± 0.02	ND	30.82 ± 0.72	20.89 ± 0.08	ND
Flavonols	F_10	Kaempferol trihexoside	3.10	C_33_H_40_O_21_	772.2062	771.1992	0.3066	[M-H]^−^	28.18 ± 0.39	ND	ND	9.07 ± 0.10	0.06 ± 0.00	ND
	F_11	Myricetin	3.27	C_15_H_10_O_8_	318.0376	317.0311	2.4073	[M-H]^−^	ND	0.09 ± 0.00	ND	ND	0.16 ± 0.00	ND
	F_12	Quercetin dihexoside	3.33	C_27_H_30_O_17_	626.1483	625.1388	−3.6122	[M-H]^−^	30.72 ± 0.13	ND	ND	27.65 ± 0.20	0.08 ± 0.00	ND
	F_13	Kaempferol hexosyl-rhamnosyl-hexoside	3.42	C_33_H_40_O_20_	756.2113	755.2041	0.1350	[M-H]^−^	49.16 ± 2.64	0.04 ± 0.00	ND	66.46 ± 4.47	1.91 ± 0.01	0.03 ± 0.00
	F_14	Myricetin hexoside	3.48	C_21_H_20_O_13_	480.0904	479.0838	1.4405	[M-H]^−^	1.72 ± 0.01	ND	ND	9.46 ± 0.09	ND	ND
	F_15	Kaempferol dihexoside	3.71	C_27_H_30_O_16_	610.1534	609.1466	0.7270	[M-H]^−^	24.18 ± 0.35	0.23 ± 0.00	ND	185.64 ± 1.81	33.69 ± 0.19	ND
	F_16	Kaempferol pentosyl-rutinoside	3.85	C_33_H_40_O_19_	740.2164	739.2105	1.8810	[M-H]^−^	2.27 ± 0.01	0.03 ± 0.00	ND	3.06 ± 0.02	0.72 ± 0.01	0.04 ± 0.00
	F_17	Quercetin hexoside-rhamnoside	4.05	C_27_H_30_O_16_	610.1534	609.1467	0.9506	[M-H]^−^	42.14 ± 0.30	0.46 ± 0.00	ND	189.71 ± 0.37	14.14 ± 0.32	0.07 ± 0.00
	F_18	Quercetin hexoside	4.32	C_21_H_20_O_12_	464.0955	463.0892	2.2038	[M-H]^−^	11.90 ± 0.43	0.15 ± 0.00	ND	250.17 ± 3.43	29.92 ± 0.51	ND
	F_19	Kaempferol rutinoside	4.38	C_27_H_30_O_15_	594.1585	593.1515	0.5894	[M-H]^−^	2.47 ± 0.02	0.12 ± 0.00	ND	123.01 ± 4.82	20.86 ± 0.07	ND
	F_20	Kaempferol pentosyl-hexoside	4.54	C_26_H_28_O_15_	580.1428	579.1327	−4.8449	[M-H]^−^	ND	ND	ND	0.33 ± 0.00	ND	ND
	F_21	Kaempferol hexoside	5.33	C_21_H_20_O_11_	448.1006	447.0945	2.7252	[M-H]^−^	0.44 ± 0.00	0.06 ± 0.00	0.02 ± 0.00	33.24 ± 0.14	6.22 ± 0.16	ND
	F_22	Quercetin rhamnoside	5.94	C_21_H_20_O_11_	448.1006	447.0944	2.5920	[M-H]^−^	ND	ND	ND	2.84 ± 0.02	ND	ND
	F_23	Kaempferol hexoside-rhamnoside	6.01	C_27_H_30_O_15_	594.1585	593.1489	−3.8024	[M-H]^−^	0.11 ± 0.00	ND	ND	3.06 ± 0.02	0.08 ± 0.00	ND
	F_24	Quercetin^+^	9.09	C_15_H_10_O_7_	302.0427	301.0350	−1.3696	[M-H]^−^	0.90 ± 0.01	ND	ND	4.41 ± 0.01	26.54 ± 0.06	ND
	F_25	Isorhamnetin	10.92	C_16_H_12_O_7_	316.0583	315.0518	2.4448	[M-H]^−^	ND	ND	ND	39.40 ± 0.10	ND	ND
	F_26	Kaempferol	11.14	C_15_H_10_O_6_	286.0477	285.0397	−2.6008	[M-H]^−^	0.37 ± 0.00	0.94 ± 0.01	0.03 ± 0.00	0.22 ± 0.00	20.65 ± 0.05	0.04 ± 0.00
	F_27	Rhamnetin	11.21	C_16_H_12_O_7_	316.0583	315.0508	−0.8097	[M-H]^−^	ND	0.81 ± 0.00	0.08 ± 0.00	ND	5.67 ± 0.28	0.24 ± 0.00
Hydroxybenzoic acids	PA_1	Gallic acid^+^	1.34	C_7_H_6_O_5_	170.0215	169.0138	−2.3862	[M-H]^−^	0.76 ± 0.02	ND	ND	ND	ND	ND
	PA_2	Hydroxybenzoic acid hexoside	1.60	C_13_H_16_O_8_	300.0845	299.0779	2.3341	[M-H]^−^	0.22 ± 0.01	ND	ND	ND	ND	ND
	PA_3	Vanillic acid	1.86	C_8_H_8_O_4_	168.0423	167.0347	−1.4459	[M-H]^−^	0.28 ± 0.00	0.86 ± 0.00	ND	ND	0.22 ± 00	ND
	PA_4	Dihydroxybenzoic acid	1.93	C_7_H_6_O_4_	154.0266	153.0187	−4.3680	[M-H]^−^	3.92 ± 0.02	0.52 ± 0.00	ND	ND	0.27 ± 0.01	ND
	PA_5	Hydroxybenzoic acid isomer I	2.46	C_7_H_6_O_3_	138.0317	137.0238	−4.2825	[M-H]^−^	ND	1.18 ± 0.01	ND	ND	2.51 ± 0.03	ND
	PA_6	Hydroxybenzoic acid isomer II	5.24	C_7_H_6_O_3_	138.0317	137.0238	−4.5771	[M-H]^−^	1.68 ± 0.03	0.32 ± 0.00	ND	ND	0.29 ± 0.00	ND
Hydroxycinnamic acids	PA_7	Caffeoylquinic acid isomer I	2.62	C_16_H_18_O_9_	354.0951	353.0884	1.6415	[M-H]^−^	8.65 ± 0.25	0.36 ± 00	ND	16.72 ± 0.31	0.58 ± 0.00	ND
	PA_8	Coumaric acid hexoside	2.73	C_15_H_18_O_8_	326.1002	325.0932	0.9335	[M-H]^−^	1.27 ± 0.01	ND	ND	0.85 ± 0.01	ND	ND
	PA_9	Ferulic acid hexoside	2.95	C_16_H_20_O_9_	356.1107	355.1038	0.9349	[M-H]^−^	1.12 ± 0.01	ND	ND	2.84 ± 0.00	ND	ND
	PA10	Sinapic acid hexoside	3.04	C_17_H_22_O_10_	386.1213	385.1150	2.4722	[M-H]^−^	0.39 ± 0.00	ND	ND	0.27 ± 0.00	ND	ND
	PA_11	Caffeoylquinic acid isomer II	3.23	C_16_H_18_O_9_	354.0951	353.0887	2.5286	[M-H]^−^	1.00 ± 0.00	0.06 ± 0.00	ND	0.39 ± 0.00	0.10 ± 0.00	ND
	PA_12	Sinapic acid	3.72	C_11_H_12_O_5_	224.0685	223.0604	−3.6818	[M-H]^−^	0.05 ± 0.00	ND	ND	ND	ND	ND
	PA_13	Coumaric acid	3.86	C_9_H_8_O_3_	164.0473	163.0394	−3.8243	[M-H]^−^	16.07 ± 0.30	ND	ND	ND	ND	ND
	PA_14	Ferulic acid	4.04	C_10_H_10_O_4_	194.0579	193.0502	−2.4580	[M-H]^−^	4.18 ± 0.02	ND	ND	3.42 ± 0.00	ND	ND
	PA_15	Coumaric acid maleate	4.65	C_13_H_12_O_7_	280.0583	279.0516	1.9362	[M-H]^−^	0.19 ± 0.00	ND	ND	0.13 ± 0.00	ND	ND
	PA_16	Coumaroylquinic acid isomer I	4.83	C_16_H_18_O_8_	338.1002	337.0933	1.1496	[M-H]^−^	0.15 ± 0.00	ND	ND	ND	ND	ND
	PA_17	Coumaroylquinic acid isomer II	5.57	C_16_H_18_O_8_	338.1002	337.0934	1.6488	[M-H]^−^	0.19 ± 0.00	ND	ND	ND	ND	ND
	PA_18	Rosmarinic acid	6.36	C_18_H_16_O_8_	360.0845	359.0776	0.9860	[M-H]^−^	3.01 ± 0.01	5.16 ± 0.03	0.02 ± 0.00	0.69 ± 0.00	1.78 ± 0.01	ND

Note: Data are shown as mean ± standard deviation of three replicates.^+^ Confirmed with commercial standards.

## Data Availability

Not applicable.

## Sample Availability

Samples of the compounds (plant material only) are available from the authors.
